# Hepatic Resection Is Safe and Effective for Patients with Hepatocellular Carcinoma and Portal Hypertension

**DOI:** 10.1371/journal.pone.0108755

**Published:** 2014-09-30

**Authors:** Jian-Hong Zhong, Hang Li, Nan Xiao, Xin-Ping Ye, Yang Ke, Yan-Yan Wang, Liang Ma, Jie Chen, Xue-Mei You, Zhi-Yuan Zhang, Shi-Dong Lu, Le-Qun Li

**Affiliations:** 1 Hepatobiliary Surgery Department, Affiliated Tumor Hospital of Guangxi Medical University, Nanning, PR China; 2 Ultrasound Department, Affiliated Tumor Hospital of Guangxi Medical University, Nanning, PR China; 3 Department of General Surgery Education, Guangxi Medical University, Nanning, PR China; 4 Hepatobiliary Surgery Department, the First Affiliated Hospital of Guangxi Medical University, Nanning, PR China; Virginia Commonwealth University, United States of America

## Abstract

**Background & Aims:**

Official guidelines do not recommend hepatic resection (HR) for patients with hepatocellular carcinoma (HCC) and portal hypertension (PHT). This study aims to investigate the safety and efficacy of HR for patients with HCC and PHT.

**Methods:**

Mortality and survival after HR were analyzed retrospectively in a consecutive sample of 1738 HCC patients with PHT (n = 386) or without it (n = 1352). To assess the robustness of findings, we repeated the analysis using propensity score-matched analysis. We also comprehensively searched the PubMed database for studies evaluating the efficacy and safety of HR for patients with HCC and PHT.

**Results:**

The 90-day mortality rate was 6.7% among those with PHT and 2.1% among those without it (*P*<.001). Patients without PHT had a survival benefit over those with PHT at 1, 3, and 5 years (96% vs 90%, 75% vs 67%, 54% vs 45%, respectively; *P* = .001). In contrast, PHT was not associated with worse short- or long-term survival when only propensity score-matched pairs of patients and those with early-stage HCC or those who underwent minor hepatectomy were included in the analysis (all *P*>.05). Moreover, the recurrence rates were similar between the two groups. Consistent with our findings, all 9 studies identified in our literature search reported HR to be safe and effective for patients with HCC and PHT.

**Conclusions:**

HR is safe and effective in HCC patients with PHT and preserved liver function. This is especially true for patients who have early-stage HCC or who undergo minor hepatectomy.

## Background

Hepatocellular carcinoma (HCC) is associated with poor prognosis. Risk factors of HCC include infection with hepatitis B virus (HBV) or hepatitis C virus (HCV) and chronic heavy alcohol consumption, which leads to liver cirrhosis [1]. Thus cirrhosis is common among HCC patients; in China, for example, it occurs in approximately 80% of HCC patients [2], [3]. Another common comorbidity of HCC is clinically significant portal hypertension (PHT), which occurs in 25–55% of patients with both HCC and cirrhosis [4]–[6]. PHT correlates with the severity of cirrhosis, and it can complicate HCC treatment by increasing the risk of perioperative hemorrhage and liver failure.

Hepatic resection (HR) is a widely used radical therapy for HCC. Although HR is often suitable for HCC patients with cirrhosis, it is widely regarded as unsuitable for HCC patients with PHT [7], [8] because of the potential for postoperative hepatic decompensation [9]. In fact, the absence of PHT is the best predictor of excellent HR outcomes [7], [8]. Guidelines of the American and European Associations for the Study of Liver Disease do not recommend HR as an option for HCC patients with PHT [10], [11]. Several studies, however, have reported that HCC patients with and without clinically significant PHT showed similar short- and long-term outcomes after HR [12]–[14]. This controversy is important to resolve because more than 25% of cirrhotic patients with HCC also present with PHT [4]–[6].

The present study aimed to address the safety and efficacy of HR for HCC patients with PHT using a population from Guangxi province of China, where the population shows the highest HCC incidence in the world [4]. Subgroup analyses were conducted based on tumor stage and extent of hepatectomy. To complement this clinical study, we searched the PubMed database for studies evaluating the efficacy of HR for HCC patients with PHT. Our goal was to clarify the indications for HR in patients with or without PHT.

## Patients and Methods

### Ethics statement

This study was approved by the Institutional Review Board of Guangxi Medical University, and it was conducted in accordance with the Declaration of Helsinki and current ethical guidelines. Written consent was given by the patients for their information to be stored in hospital databases and used for research. The hospital records data were de-identified and analyzed anonymously.

### Patients

Retrospective analysis was carried out on medical records of patients diagnosed with HCC who had been included in prospective databases at the Tumor Hospital and First Affiliated Hospital of Guangxi Medical University, the two major hospitals in Guangxi province. We planned to investigate the safety and efficacy of HR in patients with HCC and concomitant PHT by comparing survival data for patients with or without PHT. Patients were included in this analysis if they satisfied the inclusion criteria (Table 1). Curative resection was defined as any operation in which all tumors were resected macroscopically. Patients who satisfied the inclusion criteria were divided into two groups based on the presence or absence of PHT.

**Table 1:** Inclusion Criteria for Retrospective Analysis of HR Outcomes in HCC Patients with and without PHT.

	Inclusion criteria
a.	Patients underwent initial hepatic resection at one of our two liver centers
b.	Patients had cirrhosis but with Child-Pugh A liver function and underwent potentially curative hepatic resection, regardless of tumor size, tumor number, macrovascular invasion
c.	Patients showed no evidence of metastasis to the lymph nodes and/or distant metastases on the basis of preoperative imaging results and perioperative findings
d.	Patients suffered no malignancy other than HCC for 5 years prior to the initial HCC treatment

### Definitions

#### PHT

Since venous pressure is not routinely measured directly in most of the included patients, we defined PTH indirectly as the presence of gastric and/or esophageal varices detectable by endoscopy and/or computed tomography (CT), the presence of splenomegaly (pedicle rib unit >5) with a platelet count of <100,000/mm^3^, or the presence of hypertensive gastropathy [15], [16]. One of the three criterias exist implys the presence of PHT.

#### Major and minor hepatectomy

Major hepatectomy was defined as the resection of three or more Couinaud segments; minor hepactectomy, as the resection of fewer than three segments [17]. The decision whether to perform major or minor hepatectomy was based on the location and diameter of HCC and liver function tests.

#### Mortality and Morbidity

Postoperative mortality was analyzed as death within 30 and 90 days after surgery. Postoperative complications were assessed using the Clavien-Dindo classification [18].

#### Tumor Stage

Tumor stages were defined as before [4], [19].

### Diagnosis of HCC

Tumor status was assessed by ultrasonography, CT scanning, magnetic resonance imaging, and/or hepatic angiography. Since our centers did not begin using the indocyanine green test until 2010, data for this test were not used in our analysis. Vascular invasion was defined by the presence of a thrombus adjacent to the tumor in the portal and hepatic veins with vague boundaries confirmed using at least two imaging modalities [20]. In all patients, diagnosis of HCC was confirmed by histopathological examination of surgical samples.

### Treatment and follow-up

The indications for HR at our centers have already been published [4], [21], [22]. In brief, surgery was indicated when ascites and hepatic encephalopathy were absent. HR was not carried out in patients with intermediate or advanced cirrhosis and with Child-Pugh B or C liver function. Adequate remnant liver volume, as determined by volumetric CT, was 30% for HCC patients without cirrhosis, and >50% for HCC patients with chronic hepatitis, cirrhosis, or severe fatty liver. Patients who satisfied the indication for HR were treated by HR unless the patient requested other treatment modality.

Endoscopy was routinely carried out before HR. Intraoperative ultrasound was routinely performed to determine tumor location and assess the vascular anatomy of the liver. To minimize perioperative blood loss, Pringle's maneuver was carried out intermittently, each time for less than 20 minutes, with a clamp-free interval of 5 min. In most cases, the resection margin was more than 1 cm. Adequate drainage was monitored. Splenectomy, splenic embolization, and endoscopic treatments such as variceal band ligation and sclerotherapy were not performed. None of the patients underwent preoperative portal vein embolization or received portosystemic shunts before or during HR. None of the patients in our study was treated as an emergency.

After HR, all patients were periodically examined at follow-up to detect possible recurrence of HCC using liver function tests, measurement of serum alpha-fetoprotein (AFP), abdominal ultrasonography, and chest radiography. These follow-up visits were conducted every 2–3 months during the first postoperative year and every 6 months thereafter. Postoperative enhanced CT was performed every 6 months [21].

Recurrence was diagnosed on the basis of two concurring imaging techniques or the combination of increased AFP and consistent ultrasonography or CT findings, and defined as the appearance of a new lesion with radiologic features characteristic of HCC. In patients who showed recurrence or resectable extrahepatic metastasis after initial treatment, HR was performed if it was judged feasible on the basis of liver function and remnant liver volume, which were evaluated according to the same criteria as those used at the time of initial resection. If HR could not be performed because of poor liver function or other unfavorable factors, then transarterial chemoembolization, radiofrequency ablation, or sorafenib therapy were applied [4].

### Statistical analysis

All demographic and clinicopathological data had been prospectively collected in computer databases prior to this retrospective analysis. Continuous data were expressed as median (range). The statistical significance of differences in continuous data was analyzed using the Mann-Whitney *U* test, and the significance of differences between categorical data was assessed with the chi-squared test. Survival curves were estimated using the Kaplan-Meier method and compared using the log-rank test. Multivariate analysis to identify independent prognostic factors was carried out using the Cox proportional hazards model. For all tests, a two-tailed *P* value<0.05 was considered statistically significant. All statistical analyses were performed using the SPSS 19.0 statistical package (IBM, USA).

### Propensity score analysis

In order to reduce confounding and selection bias, propensity score analysis was conducted using logistic regression to create propensity scores for HCC patients with or without PHT [23]. Clinical variables already proposed as important for HR outcomes [4], [21] were assembled into a logistic regression model, which was used to generate propensity scores for each patient along a continuous range from 0 to 1. The one-to-one nearest-neighbor matching method was used to generate pairs of patients in which one had PHT and the other did not [4], [21].

### Literature review

We comprehensively searched the PubMed database using the following medical subject headings (MeSH): “*hepatocellular carcinoma* or *liver cancer* or *liver carcinoma*” and “*liver resection* or *hepatic resection* or *hepatectomy* or *surgery*” and “*portal hypertension* or *portal venous pressure*”. Manual searching of relevant references and review articles was also performed. Studies were included in our review if they (a) evaluated the efficacy of HR for primary HCC patients with PHT and provided survival data, (b) were published in English and (c) were published between January 2000 and February 2014. Studies evaluating HR to treat recurrent HCC were excluded. In the case of multiple studies based on the same population, we selected the study with the largest number of participants.

## Results

### Characteristics of the study population

Between January 2007 and December 2010, a total of 5257 patients with HCC were prospectively registered in the databases of our centers. Based on the inclusion criteria, 386 HCC patients with PHT and 1352 patients without PHT were included in the present study. Baseline demographic and clinicopathological data for the 1738 patients (Table 2) showed that more than 90% had HBV-related HCC, whereas less than 2.6% of patients were infected with HCV.

**Table 2:** Preoperative Clinicopathological Data of Patients with HCC and Child-Pugh A Liver Function who Underwent HR.

Variable	Before propensity matching			After propensity matching		
	Without PHT (n = 1352)	With PHT (n = 386)	*P* value	Without PHT (n = 224)	With PHT (n = 224)	*P* value
Median age in yr (range)	53 (19–80)	46 (17–90)	<.001	53 (19–76)	51 (19–82)	.472
Gender, M/F (%)	1221 (90.3)/131 (9.7)	344 (89.1)/42 (10.9)	.529	198 (88.4)/26 (11.6)	201 (89.7)/23 (10.3)	.650
Median tumor size in cm (range)	6.3 (2.0–18.0)	6.0 (1.0–21.0)	.060	6.0 (2.0–28.0)	6.0 (1.0–20.0)	.703
No. (%) of patients with <3/≥3 tumors	1247 (92)/105 (8)	347 (90)/39 (10)	.241	207 (92)/17 (8)	202 (90)/22 (10)	.402
No. (%) of patients +/− for hepatitis B surface antigen	1230 (91)/120 (9)	351 (91)/35 (9)	.735	199 (89)/25 (11)	199 (89)/25 (11)	.000
No. (%) of patients positive for hepatitis C antibody	31 (2.3)	10 (2.6)	.861	7 (3.1)	6 (2.7)	.778
Serum AFP, n (%)						
≥400 ng/mL	610 (45)	174 (45)	.988	101 (45)	103 (46)	.850
<400 ng/mL	742 (55)	212 (55)		123 (55)	121 (54)	
Median platelet count, ×10^3^ µ/L (range)	210 (68–528)	158 (46–533)	<.001	173 (48–600)	168 (46–533)	.504
Median prothrombin time, s (range)	12 (2–32)	14 (6–28)	<.001	14 (5–32)	14 (6–24)	.647
Median albumin, g/dL (range)	3.9 (2.3–6.2)	3.8 (2.2–5.0)	<.001	3.8 (2.8–4.9)	3.8 (2.7–5.0)	.370
Median alanine aminotransferase, U/L (range)	48 (12–185)	65 (18–260)	<.001	58 (15–185)	61 (18–220)	.879
Median total bilirubin, mg/dL (range)	0.9 (0.1–4.7)	1.2 (0.3–12.9)	<.001	1.1 (0.2–4.7)	1.1 (0.3–4.1)	.175
BCLC tumor stage, n (%)						
Early	339 (25)	116 (30)	.069	63 (28)	72 (32)	.354
Intermediate	650 (48)	174 (45)	.303	98 (44)	92 (41)	.650
Advanced (macrovascular invasion)	363 (27)	96 (25)	.521	63 (28)	60 (27)	.751
Major hepatectomy, n (%)	973 (72)	239 (62)	<.001	155 (69)	146 (65)	.370
30-day mortality, n (%)	13 (1.0)	9 (2.3)	.051	4 (1.8)	4 (1.8)	.000
90-day mortality, n (%)	28 (2.1)	26 (6.7)	<.001	7 (3.1)	6 (2.1)	.780
Postoperative complications, n (%)	311 (23)	120 (31)	.004	47 (21)	63 (28)	.080
Median survival time in mos. (range)	69 (1–152)	48 (1–132)	<.001	64 (1–129)	47 (1–106)	<.001

AFP, α-fetoprotein; BCLC, Barcelona Clinic Liver Cancer; PHT, portal hypertension.

Patients with PHT had worse liver function, i.e. lower platelet count and albumin concentration, longer prothrombin time, and higher concentrations of alanine aminotransferase and bilirubin (all *P*<.05). More patients with PHT underwent minor hepatectomy (*P*<.001).

Anatomic HR was the preferred surgical procedures. Most patients with multiple tumors underwent two or more concomitant HR. The feasibility of anatomic HR was significantly lower in the PHT group than in the non-PHT group. However, the surgical time and blood loss were similar between the two groups.

### Morbidity and mortality in the entire study population

Patients with PHT had a significantly higher morbidity rate (31%) than those without PHT (23%, *P* = .004). However, most of the complications were grade I or II (Table 3). Patients with or without PHT showed similar 30-day mortality (2.3% vs 1.0%, *P* = .051), while patients with PHT showed significantly higher 90-day mortality (6.7% vs 2.1%, *P*<.001).

**Table 3:** Postoperative Complications in HCC Patients with or without PHT After HR, Assessed Using the Clavien-Dindo Classification Scheme.

Grade	No. (%) of patients		*P* value
	Without PHT (n = 1352)	With PHT (n = 386)	
I	252 (18.6)	104 (26.9)	.007
II	181 (13.4)	67 (17.4)	
III-a	81 (6.0)	30 (7.8)	
III-b	55 (4.1)	21 (5.5)	
IV-a	30 (2.2)	30 (7.8)	
IV-b	51 (3.8)	16 (4.1)	
V	28 (2.1)	26 (6.7)	

Among patients who underwent minor hepatectomy, 90-day mortality was similar between those who had PHT (4/147, 2.7%) and those who did not (2/379, 0.5%; *P* = .07). Among patients who underwent major hepatectomy, 90-day mortality was higher among those with PHT (22/239 or 9.2% vs 26/973 or 2.7%; *P*<.001).

### Survival analysis in the entire study population

**Figure 1:**
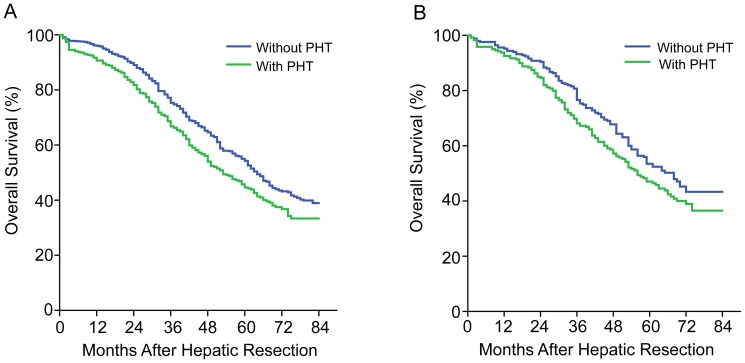
Overall survival curves of the population undergoing HR for HCC with and without PHT. (A) Total population (*P*<.001). (B) Propensity score-matched population (*P* = .061).

During a median follow-up of 39.3 months (range, 1–152.4), 198 patients with PHT (51.3%) died, compared to 549 patients without PHT (40.6%). These included 582 (78%) patients died of HCC recurrence, 38 (5%) died of liver failure without evidence of recurrence, and 127 (17%) died of other diseases. Median survival time was 69.2 months in patients without PHT, compared to 48.1 months in patients with PHT (*P*<.001). Overall survival (OS) at 1, 3, and 5 years was 90%, 67%, and 45% for patients with PHT, and 96%, 75%, and 54% for patients without (*P* = .001; Fig. 1A).

However, cumulative recurrence rates were similar between the PHT group and non-PHT group at 1 year (30% vs 28%), 3 years (50% vs 46%), and 5 years (60% vs 55%; *P* = .11). Moreover, the sizes and number of recurrent tumors, and the proportion of patients with recurrence in whom a second HR was indicated were similar between the two groups (Table 4). OS in each group was better among the patients with recurrence who underwent a second HR as compared with those who did not.

**Table 4:** Characteristics and treatment modalities for recurrence.

Variable		Without PHT (n = 1352)	With PHT (n = 386)	*P* value
Number of recurrence		798 (59%)	248 (64%)	.064
Median tumor size in cm (range)		1.4 (0.7–3.8)	1.3 (0.8–3.6)	.156
Site of recurrence	Extrahepatic	200 (25%)	22 (9%)	<.001
	Intrahepatic	598 (75%)	226 (91%)	
Tumor number of intraheaptic recurrence	Single	310 (41%)	94 (38%)	.396
	Multiple	447 (59%)	154 (62%)	
Treatment for recurrence	Second resection	311 (39%)	87 (35%)	.276
	Transarterial chemoembolization	479 (60%)	156 (63%)	
	Radiofrequency ablation	8 (1%)	5 (2%)	

Univariate analysis identified the following prognostic risk factors in the total population: tumor number ≥3, serum AFP ≥400 ng/mL, serum albumin <4 g/dL, serum alanine aminotransferase >80 U/L, serum bilirubin >1.2 mg/dL, macrovascular invasion, major hepatectomy, and PHT. All these factors, except serum albumin, were also identified in multivariate Cox proportional hazard modeling as independent predictors of poor prognosis (Table 5).

**Table 5:** Multivariate Analysis of Clinicopathological Factors Predictive of Poor Overall Survival.

Variable	Before propensity matching			After propensity matching		
	Hazard ratio	95% confidence interval	*P* value	Hazard ratio	95% confidence interval	*P* value
Tumor number≥3	1.543	1.233–1.931	<.001	1.543	1.045–2.277	.029
α-fetoprotein≥400 ng/mL	1.321	1.171–1.491	<.001	1.263	1.015–1.572	.037
Albumin<4 g/dL	0.880	0.776–1.026	.120	0.902	0.715–1.138	.386
Alanine aminotransferase>80 U/L	1.196	1.007–1.421	.041	0.978	0.756–1.265	.866
Bilirubin>1.2 mg/dL	1.161	1.010–1.334	.035	0.950	0.757–1.191	.654
Macrovascular invasion	1.541	1.341–1.769	<.001	1.415	1.100–1.821	.007
Major hepatectomy	1.232	1.142–1.662	.021	1.025	1.080–1.523	.046
Portal hypertension	1.830	1.554–2.154	<.001	1.609	1.285–2.013	<.001

### Subgroup analysis by Barcelona group tumor stage

**Figure 2:**
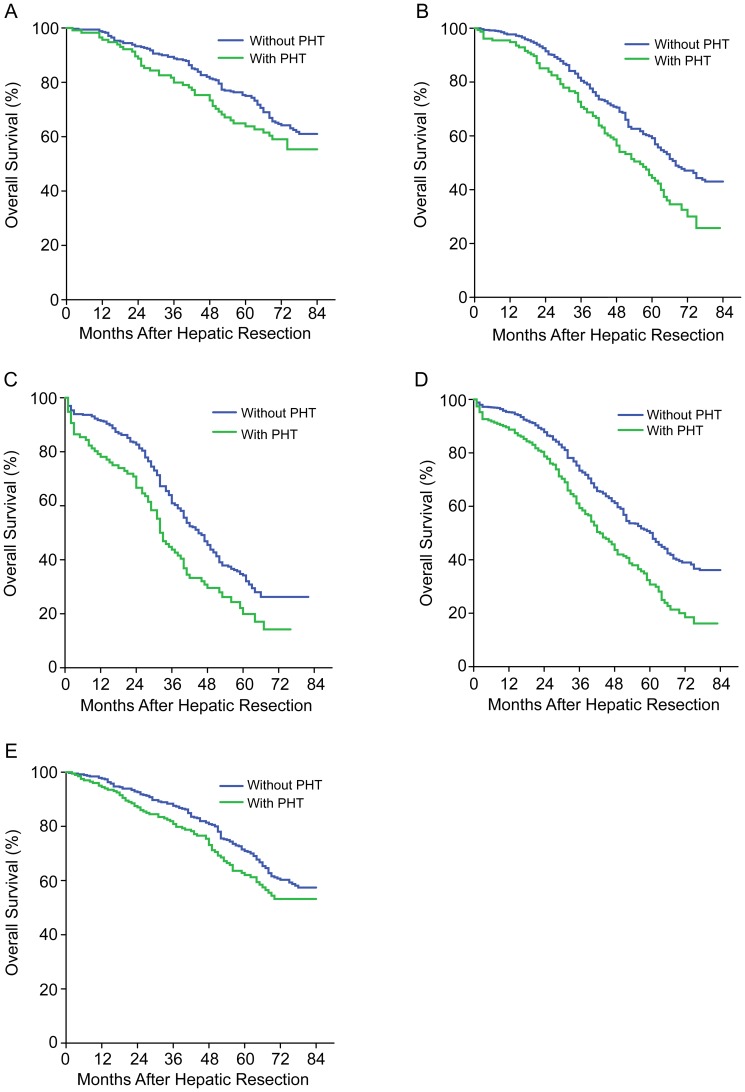
Overall survival curves of subgroup analysis. (A) Early-stage HCC (P = .108). (B) Intermediate-stage HCC (P = .004). (C) Advanced-stage HCC (P = .001). (D) Major hepatectomy (P<.001). (E) Minor hepatectomy (P = .064).

We examined whether the efficacy of HR depended on tumor stage. Among the 455 patients with early-stage HCC (26% of the total), patients with and without PHT had similar OS at 1 year (96% vs 99%), 3 years (80% vs 89%), and 5 years (63% vs 75%) (*P* = .108; Fig. 2A). However, among the 824 patients with intermediate-stage HCC (47%), patients with PHT had significantly worse OS than those without PHT at 1 year (94% vs 97%), 3 years (69% vs 77%), and 5 years (45% vs 56%) (*P* = .004; Fig. 2B). Similar results were observed among the 459 patients with advanced-stage HCC (27%), which was defined as HCC with macrovascular invasion: patients with PHT had significantly worse OS at 1 year (78% vs 91%), 3 years (43% vs 60%), and 5 years (20% vs 34%) (*P* = .001; Fig. 2C).

### Subgroup analysis by major or minor hepatectomy

Of our 1738 patients, 1212 (70%) underwent major hepatectomy. Their OS was significantly lower if they had PHT: 88% vs 95% (1 year), 59% vs 73% (3 years), and 30% vs 50% (5 years) (*P*<.001; Fig. 2D). However, among the 526 (30%) patients who underwent minor resection, PHT was not associated with worse OS: 1 year, 95% vs 98%; 3 years, 77% vs 87%; and 5 years, 59% vs 71% (*P* = .064; Fig. 2E).

### Characteristics and survival analysis of propensity-matched patients

Propensity score analysis based on variables associated with prognosis identified 224 matched pairs of patients. When only these pairs were considered, no significant baseline difference between the two groups was observed (Table 2). Patients selected in the propensity-matching model with PHT did not show significantly worse survival than did those without PHT (*P* = .061; Fig. 1B). The 1, 3, and 5 years OS was 92%, 68%, and 46% for patients with PHT, and 95%, 76%, and 52% for patients without PHT. Cumulative recurrence rates at 1 (26% vs 29%), 3 (45% vs 51%), and 5 years (56% vs 65%) were similar between patients with and without PHT (*P* = .21).

Patients with and without PHT had similar morbidity (28% vs 21%, *P* = .08), 30-day mortality (1.8% vs 1.8%, *P* = 1.00), and 90-day mortality (2.1% vs 3.1%, *P* = .78). The multivariate Cox proportional hazards model identified tumor number (≥3), serum AFP (≥400 ng/mL), macrovascular invasion, major hepatectomy, and PHT as mortality risk factors (Table 5).

### Literature review

A comprehensive search of the PubMed database identified 9 studies that satisfied the inclusion criteria; they examined HCC patients with and without PHT who were treated by HR in Italy [5], [12]–[14], [24], Korea [25], and Japan [26]–[28] and who had been recruited between 1982 and 2012 (Table 6). Hospital mortality at 3 months was less than 5% for patients without PHT, while it was more than 6% for patients with PHT. Interestingly, the study [28] that recruited patients starting in 1982 reported 1-month mortality as high as 12.9%, while the study [12] that recruited patients starting in 1985 reported 2-month mortality of 11.1%. The downward trend of mortality reflects the improvement of surgical techniques.

**Table 6:** Characteristics and Outcomes of Patients with PHT Following Curative Resection of HCC, Based on a Literature Review.

Study	Patient origins	Recruitment period	N (no PHT/PHT)	Without PHT (%)				With PHT (%)			
				Mortality	1 yr OS	3 yr OS	5 yr OS	Mortality	1 yr OS	3 yr OS	5 yr OS
Capussotti et al^12^	Italy	1985–2003	118/99	5.1 (2 m)	76	62	40	11.1 (2 m)	71	45	29
Choi et al^24^	Korea	1996–2006	53/47	1.9 (1 m)	92	79	79	6.4 (1 m)	77	48	38
Cucchetti et al^5^	Italy	1997–2007	152/89	-	-	73	62	-	-	62	52
Giannini et al^13^	Italy	1987–2008	70/42	-	98	82	60	-	100	87	75
Hidaka et al^25^	Japan	1997–2009	129/48*	-	92	78	64	-	73	49	31
Ishizawa et al^26^	Japan	1994–2004	250/136	-	-	81 and 62†	71 and 31†	-	-	71 and 59†	56 and 41†
Kawano et al^27^	Japan	1982–2003	103/31	8.7 (1 m)	-	-	48	12.9 (1 m)	-	-	70
Ruzzenente et al^14^	Italy	1995–2008	91/44	3.3 (3 m)	-	68	61	13.6 (3 m)	-	49	45
Santambrogio et al^23^	Italy and France	1997–2012	160/63	1.9 (3 m)	-	80	65	6.3 (3 m)	-	66	48
This study	China	2007–2010	1352/386	2.1 (3 m)	98	81	60	6.7 (3 m)	91	72	37

* patients were divided into a group with high portal venous pressure (≥20 cmH_2_O) and a group with low portal venous pressure (<20 cmH_2_O);

† Child-Pugh class A cirrhosis and Child-Pugh class B cirrhosis.

The 5-year OS after HR ranged from 29% to 75% for patients with HCC and PHT, and 31% to 79% for patients without PHT (Table 6). In 6 studies, median survival was significantly longer for patients without PHT [5], [12], [14], [24]–[26], while 3 studies found similar median survival between the two patient groups [13], [27], [28]. All 9 studies identified in our review explicitly concluded that PHT should not be considered an absolute contraindication for HR in cirrhotic patients [5], [12]–[14], [24]–[28].

## Discussion

Historically HR has been applied with caution to HCC patients because of concerns about morbidity and mortality; indeed mortality rates with HR used to exceed 10%. However, recent improvements in surgical technique and perioperative care have improved HR outcomes even as the procedure has been extended to more high-risk patients; some major liver centers now report hospital mortality below 5% [29], [30]. Nevertheless selecting patients for HR remains important. For example, PHT may increase the risk of perioperative hemorrhage, impair liver regeneration, and increase the risk of liver failure, leading the American and European Associations for the Study of Liver Disease to classify PHT as a contraindication to HR. These guidelines are based on Barcelona group studies of very small sample size [7], [8], and they conflict with larger studies showing that HCC patients with and without clinically significant PHT show similar short- and long-term outcomes after HR [12]–[14]. Given the high frequency of PHT among HCC patients in China [4] and elsewhere [11], it is important to resolve this lack of consensus on whether HR is safe and effective for such patients.

In this clinical study of a large cohort of patients from a region in which more than 77% of HCC is associated with cirrhosis [4], OS for HCC patients with PHT after HR was 67% at 3 years and 45% at 5 years. OS at 1, 3, and 5 years for both the two groups was similar to survival results reported by studies that we identified in a comprehensive review of PubMed. In contrast to our results, some of those previous studies found similarly good OS in patients with and without PHT (Table 6).

Our previous study reported that HR of patients with intermediate- and advanced-stage HCC and PHT gave better survival than transarterial chemoembolization [4], and another study reported that HR was associated with longer survival and time to recurrence than radiofrequency ablation [31]. For Child-Pugh A cirrhotic patients with a single HCC up to 5 cm, HR offers a similar 5-year OS to liver transplantation, even for patients with PHT [32], [33]. However, the liver donation is shortage in most countries. In this way, we take a different position from that of the American and European guidelines, recent seminars [11], [34], [35] and consensus statements of the Asian Pacific Association for the Study of the Liver [36], the Japan Society of Hepatology [37], and the American Hepato-Pancreato-Biliary Association [38].

Though we argue for expanding HR to HCC patients with PHT, we emphasize that patient selection remains critical for success. The two most important selection criteria in our study were preoperative Child-Pugh A liver function and >50% postoperative residual liver volume. Careful application of these rigorous selection criteria may explain why patients with and without PHT showed similar OS after being stratified by Barcelona group tumor stage or extent of hepatectomy. It may also explain why 90-day mortality was similar between patients with and without PHT who underwent minor resection. Our findings suggest that as long as appropriate inclusion criteria are strictly applied, there is no absolute contraindication of HR for treating HCC patients with PHT.

Indeed, we suggest that our strict selection process explains why the mortality of our population of patients with PHT and early-, intermediate- or advanced-stage HCC was 6.7% (26/386), whereas the mortality of an earlier group of patients we studied with PHT and intermediate- or advanced-stage HCC was 10.3% (17/166) [4]. The present cohort was recruited over the period from 2007 to 2010, while the earlier cohort was recruited from 2000 to 2007. This suggests gradual improvement in mortality over time. In fact, some liver centers have reported zero perioperative mortality during HR of HCC patients [30], [39]. These findings provide further support for our suggestion that, largely due to improvements in surgical technique and medical care, HR is safe for HCC patients with PHT, especially for those with early-stage HCC and those undergoing minor resection.

Some studies suggest that PHT is an independent risk factor of morbidity and mortality [4], [8], [40], [41]. The main risks of HR in patients with PHT are liver failure, varices rupture, and coagulation disorders caused by thrombocytopenia. Patients with PHT tended to have poorer preoperative liver function than those without PHT (Table 2), even though all our patients had Child-Pugh A liver function. Patients with PHT showed a higher morbidity rate (31%) than those without PHT (23%); nevertheless, most postoperative complications were Clavien-Dindo grade I or II. Of the 26 patients who died within 90 days of HR in the PHT group, 15 underwent major hepatectomy and died from postoperative liver failure, 5 died from varices rupture, and 3 died from thrombocytopenia. The remaining 3 patients had concurrent liver failure and varices rupture. Therefore we recommend selecting patients with PHT carefully for major hepatectomy, especially when preoperative liver function is unsatisfactory, regardless of whether the expected remnant liver volume is more than 50%. Major hepatectomy may, in fact, aggravate PHT because it reduces the hepatic parenchymal mass. For HCC patients whose PHT poses a problem for HR, alternative therapies or a reduction in the extent of hepatectomy may be preferable.

In HCC patients with severe gastric and/or esophageal varices, it may be possible to reduce the rate of varices rupture by using preoperative endoscopic treatments and/or splenectomy with or without devascularization of the abdominal esophagus and the upper part of the stomach [27].

Platelet count less than 150,000/mm^3^ is independently associated with increased major complications and mortality [15], [29]. Preoperative platelet transfusion was performed in the 3 patients who ultimately died from thrombocytopenia; these outcomes may reflect the difficulties of controlling coagulation disorders effectively once the patient has been discharged. In our experience, HR should be selected with caution when preoperative platelet counts are less than 75,000/mm^3^.

Preoperative hepatic venous pressure gradient (HVPG) was not routinely assessed in patients with PHT. HVPG correlates with the severity of PHT [42]. Elevated HVPG (median levels of 11–12 mmHg) is associated with postoperative liver dysfunction and mortality after HR in patients with HCC and cirrhosis [38], [42], [43], independently of esophageal varices, splenomegaly and thrombocytopenia [39]. In one of those studies [40], 26 patients were diagnosed with PHT using indirect methods, but only 18 of these had an HVPG ≥10 mmHg, which Barcelona group studies[7], [8] have proposed as the cut-off when HPVG becomes an independent risk predictor of liver dysfunction. This suggests that some patients diagnosed with PHT by indirect methods do not fulfill the Barcelona group cut-off, highlighting the need for direct measurement of preoperative HVPG measurement in patients with clinically significant PHT.

Our multivariate Cox modeling to identify prognostic factors in HCC patients with or without PHT led to similar conclusions as previous studies: patients with high preoperative AFP [4], alanine aminotransferase [21] and bilirubin levels [8], multinodularity [44], macrovascular invasion [4], history of major resection [45], and PHT [41] had significantly worse outcomes than did other patients after HR. However, when this analysis was repeated using propensity-matched patient pairs, elevated alanine aminotransferase and bilirubin were not identified as risk factors. Elevated AFP can be a useful prognostic marker in patients with HCC who do not show any well-established risk factors. Multinodularity and macrovascular invasion are risk factors presumably because they have been linked to the emergence of *de novo* tumors and HCC recurrence, respectively [34], [46], [47]. Our finding of PHT as an independent risk predictor may explain the high 90-day mortality (6.7%) among patients with PHT. When we repeated the multivariate analysis after excluding 73 patients who survived fewer than 20 months after surgery, PHT remained a significant risk factor. PHT may be such a strong risk factor at least in part because it so often co-occurs with severe cirrhosis, which on its own is an independent risk factor for carcinogenesis.

Even though our patients came from the region with the highest incidence of HCC in the world [4], we believe our results are generalizable to other populations because our findings are consistent with an extensive literature review in which we identified studies of patient populations in several other countries.

## Conclusions

Our clinical studies at two large liver care centers together with our literature review strongly suggest that HR is safe and effective for HCC patients with PHT and preserved liver function, especially for those with Barcelona group early-stage HCC and those who undergo minor hepatectomy.
